# A Practical Treatise on the Diseases of Children

**Published:** 1846-10

**Authors:** 


### ARTICLE VI.

*A Practical Treatise on the Diseases of Children.* By JAMES MILMAN COLEY, M. D., Member of the Royal College of Physicians, London, &c. &c. Philadelphia: Ed. Barrington & Geo. D. Haswell. 1846. 8vo. pp. 414. (From the Publishers.)

This work is one of the publications issued in the Select Medical Library, edited by John Bell, M. D. That it should have been selected for publication, by the erudite and experienced editor of this well known periodical, is in itself sufficient recommendation to the work. The author professes to give a comprehensive view of the diseases of children, with a view to render his work one of reference, and, we think, has succeeded well in his object. Upon some points of pathology and treatment there is some originality, and as regards treatment it is simplified to a degree which we cannot but admire. Our perusal of the work has not been as complete as we could wish, from the fact that it arrived too late for an extended notice. Should our present good opinion of it continue on more accurate examination, we may, in our next number present our readers with a review embracing extracts from its more important portions. J. V. Z. B.

